# Budgetary impact analysis of buprenorphine-naloxone combination (Suboxone^®^) in Spain

**DOI:** 10.1186/2191-1991-2-3

**Published:** 2012-03-29

**Authors:** Jose Martinez-Raga, Francisco Gonzalez-Saiz, Julian Oñate, Itziar Oyagüez, Eliazar Sabater, Miguel A Casado

**Affiliations:** 1 Unidad Docente de Psiquiatría y Psicología Médica, University of Valencia Medical School and Unidad de Conductas Adictivas de Gandía, Agencia Valenciana de Salut, Valencia, Spain; 2 Unidad de Salud Mental Comunitaria Villamartin. UGC Salud Mental Hospital de Jerez, Servicio Andaluz de Salud, Spain; 3 Drogodependencias y Salud Mental, Murcia, Spain; 4 Pharmacoeconomics and Outcomes Research Iberia, C/de la Golondrina 40A. Madrid 28023, Madrid, Spain

**Keywords:** Buprenorphine-naloxone, Methadone, Budgetary impact, Opioid dependence, Spain

## Abstract

**Background:**

Opioid addiction is a worldwide problem. Agonist opioid treatment (AOT) is the most widespread and frequent pharmacotherapeutic approach. Methadone has been the most widely used AOT, but buprenorphine, a partial μ-opiod agonist and a κ-opiod antagonist, is fast gaining acceptance. The objective was to assess the budgetary impact in Spain of the introduction of buprenorphine-naloxone (B/N) combination.

**Methods:**

A budgetary impact model was developed to estimate healthcare costs of the addition of B/N combination to the therapeutic arsenal for treating opioid dependent patients, during a 3-year period under the National Health System perspective. Inputs for the model were obtained from the specialized scientific literature. Detailed information concerning resource consumption (drug cost, logistics, dispensing, medical, psychiatry and pharmacy supervision, counselling and laboratory test) was obtained from a local expert panel. Costs are expressed in euros (€, 2010).

**Results:**

The number of patients estimated to be prescribed B/N combination was 2,334; 2,993 and 3,589 in the first, second and third year respectively. Total budget is €85,766,129; €79,855,471 and €79,137,502 in the first, second and third year for the scenario without B/N combination. With B/N combination the total budget would be €86,589,210; €80,398,259 and €79,708,964 in the first, second and third year of the analyses. Incremental cost/patient comparing the addition of the B/N combination to the scenario only with methadone is €10.58; €6.98 and €7.34 in the first, second and third year respectively.

**Conclusion:**

Addition of B/N combination would imply a maximum incremental yearly cost of €10.58 per patient compared to scenario only with methadone and would provide additional benefits.

## Background

Opioid abuse remains a serious public health problem worldwide, notably in Asia and Europe. Worldwide prevalence of opioid use has been estimated at 0.4% [1]. In Spain, the most recent available data indicated that 0.8% of the population aged 15-64 used opioids in 2007 [2]. Opioid addiction is associated with great economic burden [3], as well as various and severe health problems, including an increased risk for HIV/AIDS and viral hepatitis B and C infection, generally as a consequence of intravenous drug use. Mortality rates are high as well, particularly among individuals 15-34 years of age [4]. Mortality in dependent heroin users is between 6 and 20 times that expected for the general population of the same age and gender. As a consequence, in many countries, opioid use constitutes the main cause of drug-related deaths [1].

Pharmacological interventions for heroin dependence aim primarily at maximizing treatment retention, attaining long-term abstinence and minimizing the risk of returning to the previous pattern of drug abuse following safe and efficient suppression of opioid withdrawal symptoms [5,6]. Different types of medications are used in the management of opioid dependent patients, including opioid agonists and partial agonists, opioid antagonists and alpha (2)-adrenergic agonists [6-8]. Agonist opioid treatment (AOT) is the most common intervention for heroin dependence [9]. Methadone, as part of an AOT program, is the most widely used and well researched pharmacotherapy for heroin dependent patients since the 1960s [10]. However, other agonists alone or in combination with antagonist compounds are increasingly used [9].

Buprenorphine, a partial μ-opiod receptor agonist and a κ-opiod receptor antagonist, is fast gaining acceptance among addiction specialists and patients [10-12], partly due to methadone, as the sole available AOT in most countries fails to meet the specific treatment needs of all opioid-dependent individuals who might benefit from medication. This may help to explain why a significant proportion of these patients remain untreated [13]. Solid evidence has shown the efficacy of buprenorphine for treating heroin addiction [8,14-16]. A buprenorphine/naloxone (B/N) combination (Suboxone^®^, Reckitt Beckinser Pharmaceuticals Limited) was approved by the European Medicines Agency (EMA) in September 2006 as oral substitution treatment for opioid drug dependence within a framework of medical, social and psychological treatment [17]. The rationale for developing a new compound, combining naloxone with buprenorphine was to minimize the risk of intravenous misuse. Injection of naloxone would precipitate opioid withdrawal in opioid-dependent patients, but taken sublingually, as prescribed, the bioavailability of naloxone is negligible, therefore allowing for the patient to benefit from effects of buprenorphine alone [18,19]. As a result of the reduced risk for diversion and misuse there is less need for direct close supervision of medication during OST, less resources involved therefore allowing for a reduction in associated costs and for treatment to become more accessible [20]. In addition, there is evidence that buprenorphine/naloxone is an effective and well-tolerated treatment for medically assisted opioid withdrawal when the dosage is titrated to achieve good control of withdrawal symptoms [21,22].

Additionally to the primary outcomes on efficacy/effectiveness and safety, the results provided in economic evaluations (cost-effectiveness and budgetary impact) could be a crucial source to be used by health care provider for the purpose of decision making [23]. Budgetary impact studies are a direct tool for helping decision makers to perform a better allocation of available resources, mainly in the present global situation of cost constraint we are living.

The aim of this study was to assess the budgetary impact in Spain of buprenorphine-naloxone (B/N) combination (Suboxone^®^) as a newly approved pharmacotherapy for opioid dependence, specifically compared to methadone the only widely available medication for heroin addiction.

## Methods

### Model

**Figure 1:**
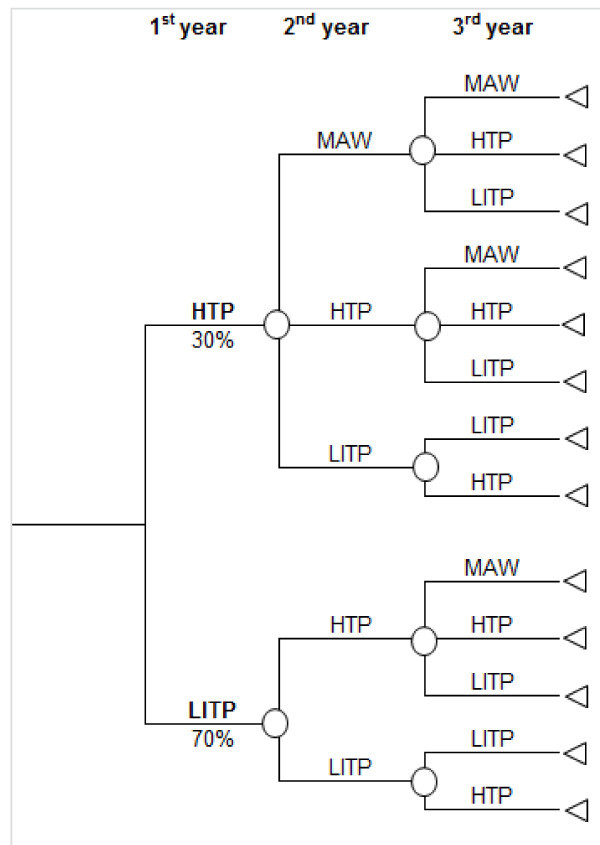
**Diagram representation of the decision tree**.

A previous published [24] budgetary impact model using Microsoft Excel 2003 following the international recommendations [25-28] has been updated to estimate healthcare costs of the approval of B/N combination as a pharmacotherapy for opioid dependence. A decision tree [29] based on an expert panel consensus was devised to describe progress-over-time of patients in AOT. (Figure 1) Simulation of events and outcomes within the therapeutic strategies assessed were represented with as many arms as possible available options for the different populations taken into account. For each possible outcome or decision-tree-arm and for each treatment option, the corresponding probabilities of transition were estimated. The transition probabilities used in the simulation, as obtained from a literature review [24], are summarized in Table 1. Opinion of the expert panel was sought for missing or controversial information. The initial probabilities for the first year were maintained in subsequent cycles.

**Table 1:** Transition probabilities

From:	MAW	HTP		LITP
			
To:		Methadone	B/N	
**MAW**	50.0%	5.0%	18.0%	0.0%

**HTP**	35.0%	75.0%	63.0%	5.0%

**LITP**	15.0%	20.0%	19.0%	95.0%

### Population

Eligible population was obtained from estimates of National Program on Drugs [30]. Approval of B/N have not increased the total annual population in AOT, consequently the number of patients remained unchanged throughout the simulation.

In concordance with previous studies three target population groups were identified among the total eligible population [24]:

1) Patients needing medically assisted withdrawal (MAW) program prior to entering a relapse prevention program, therefore not in AOT.

2) Patients in high threshold programs (HTP). These included those individuals without any physical or psychological impairment, and despite showing good adherence to AOT have difficulties in staying abstinent. These patients require high levels of supervision.

3) Patients in low-intermediate threshold program (LITP) were those with some physical and/or psychological impairment and poor adherence to AOT. These patients have less supervision and more commonly are poly-substances abusers.

In the base case of the model, the first year of the simulation begins with no patients on MAW stage. According the expert panel opinion, in the first year 30.0% of patients are HTP and 70.0% are LITP.

### Treatment options

Two pharmacotherapies were considered in the analysis: methadone and B/N combination. Although the opioid antagonist naltrexone was also available as a treatment option for relapse prevention for heroin dependent subjects, this medication was excluded from the analysis due its limited acceptance among both patients and clinicians [31] and the lack of sufficient evidence supporting its use as a maintenance therapy for opioid dependence [32].

An expected 10.0%, 15.0% and 20.0% of patients entering B/N combination were considered in the model in the first second and third year, respectively, in the HTP group. It was assumed that all patients in LITP would remain on methadone treatment. Two scenarios were compared in the analysis: a situation with 100.0% of patients in AOT treated with methadone versus an alternative option considering annual gradual increases of patients entering B/N combination.

### Time horizon, perspective and discounting

The analysis was performed with a 3-year time projection with 3 cycles of 1 year each within the context of the National Health System. However, considering that in Spain addiction treatment programs and services are organized and developed by Autonomous Regional Governments, results are shown by Autonomous Regions, as well.

As stated in the good practices of the International Society for Pharmacoeconomics and Outcomes Research (ISPOR) [26], budget impact analyses present financial streams over time, and it is not necessary to discount the costs.

### Resources and costs

The following resources and treatment components were identified and quantified:

1) *Drug costs*: Pharmacological costs of the pharmacotherapies evaluated that were calculated based on defined daily dose as established by the World Health Organization [33] and expert panel opinion. An average daily dose of 8 mg per patient was considered for B/N combination, and of 60 mg for methadone (independently of whether patients were HTP or LITP).

2) *Logistic costs*: Distribution and production resources were considered. These included a series of procedures generally performed by an accredited pharmacist such as collecting and storing methadone with adequate safety measures, transporting the methadone to central pharmacies for individual methadone hydrochloride dose preparation, subsequent distribution to the dispensing centres, and finally collection of unused doses. An average of four minutes per methadone dose for distribution and 2 minutes per methadone dose for production were considered for the purpose of cost estimation. No logistics resources were involved in B/N combination.

3) *Dispensing*: Five minutes of nursing staff for dispensing methadone to patients was considered in the analysis. Methadone dispensing twice a week was taken into account in HTP patients for the first year (104 dispensations) and once a week for the second and third year (52 dispensations per year). Once a week dispensing was considered for LITP patients. No dispensing was required in the case of B/N combination treatment. In addition, it was assumed that 36 dispensation (associated with non-AOT drugs) per year are needed in MAW patients.

4) *Medical, psychiatry and pharmacy staff involvement*: Medical supervision (15 minutes) is required for patients in AOT. Ten visits by year in MAW and HTP patients and 6 visits by year in LITP. For psychiatric care 10 sessions (25 minutes/session) were assumed in MAW and first year of treatment in HTP. Ten sessions by year was also assumed for 2^nd ^and 3^rd ^year in HTP methadone treated patients. Five sessions by year for 2^nd ^and 3^rd ^year in patients treated with B/N combination. No psychiatric care for LITP patients was included. Pharmacy supervision (1 minute) is estimated to be done 36 times by year in MAW patients, and daily in HTP and LITP.

5) *Counselling*: Twenty-five minutes sessions with specialist addiction of psychologist were also included in the analysis. A total of 20 sessions/year for MAW and first year of treatment for HTP patients was assumed, whilst for each of the second and third year the number of estimated sessions was 10 for methadone treated patients and 5 for patients treated with B/N combination. In addition, the assumption was made that LITP patients would not be involved in specialist psychological counselling. Social and employment rehabilitation care (15 minutes per session) provided by social workers to individuals in AOT was also taken into account. (See Table 2 for detailed social worker assistance)

**Table 2:** Resource consumption

			HTP			LITP	
		
Resource	MAW	Methadone 1^st ^year	Methadone _2_^nd ^_& 3_^rd ^year	B/N 1^st ^year	B/N _2_^nd ^_& 3_^rd ^year	Methadone	B/N
**Distribution**		365	365				

**Production**		365	365			365	

**Dispensing**	36	104	52			52	

**Supervision**							

drug-pharmacy	36	365	365			365	

medical/clinical						6	6

psychological	20	20	10	20	5		

social worker	15	10	10	10	5	6	6

psychiatric	10	10	10	10	5		

**Monitoring (analytical controls)**	36	12	12	12			

6) *Laboratory test*: Urine toxicology drug screenings were also included in the analysis.

All costs are expressed in euros (€, 2010). Resource unitary costs (Table 3) were collected from literature [24] and a Spanish costs database updated to 2010 value with Consumer Price Index [34]. Concerning pharmacological costs, the ex-factory prices of medications were considered. The 7.5% reduction of ex-factory price required by Health Authorities was applied to B/N combination [35].

**Table 3:** Unitary costs (€, 2010)

Resource	Cost (€, 2010)
**Drugs**	

Methadone	531.80 € per methadone kg

Buprenorphine/Naloxone combination (Suboxone^®^, 8/2 mg, 7 tablets)	2.37€ per tablet

**Logistics**	

Distribution	0.17€/min

Production	0.49€/min

Dispensing	

**Nurse**	0.28€/min

**Supervision**	

Drug-pharmacy	0.49€/min

Medical/clinical	0.49€/min

Psychological	0.49€/min

Social worker	0.28€/min

Psychiatric	0.49€/min

**Monitoring (analytical controls)**	3.81€/test

### Sensitivity analyses

One-way sensitivity analyses were performed to test the robustness of the model. Base case values were modified for the following parameters:

-Transition probabilities: The same values applied for methadone were also assumed for B/N combination.

-Patients commencing B/N combination: In Spain there is only an Autonomous Region (Murcia) where B/N combination is fully financed by the Regional Health Care System with a free access to the drug. A sensitivity analysis with the proportion of patients starting B/N combination treatment over the previous three years available for this region was performed (0.97%, 2.57% and 3.83%) [36]

-Initial distribution of patients: Influence of 100% of patients in LITP or 100% of patients in HTP was tested.

-Resource costs: Differences of ± 10% in cost per minute of resources were tested.

## Results

### Treatment population: patients on AOT programs

According to National Health estimates of the Spanish National Program on Drugs [30], a total of 77,811 patients were calculated to be enrolled in AOT.

According to patient distribution within each target group and the projection of the percentage of specific AOT medication use over the three years of the study period, the number of patients expected to be treated with B/N combination during the first, second and third year of the study was 2,334; 2,993 and 3,589, respectively. The simulation in the defined base case begins with 30.0% of the total population in HTP. In the scenario without B/N combination, a total of 1,167 patients progressed to MAW in the 2^nd ^year and 1,595 in 3^rd ^year. Addition of B/N combination was associated with 1,466 and 2,114 MAW patients in the first and second year.

### Budgetary impact

Total budgetary impact was €85,766,129, €79,855,471 and €79,137,502 in first, second and third year, respectively, for the scenario without B/N combination. In contrast, with the availability of B/N combination the total budgetary impact would be €86,589,210, €80,398,259 and €79,708,964 in the first, second and third year of the analyses, respectively (Table 4). Detailed results by Spanish Autonomous Regions are shown in Table 5.

**Table 4:** Budgetary impact results (€, 2010)

		Scenario without B/N combination	Scenario with B/N combination	Difference (€) with B/N vs. without B/N
**1st year**	MAW	1,724,290	2,312,217	587,927

	HTP	28,428,367	28,541,622	113,255

	LITP	55,613,472	55,735,372	121,900

**2^nd ^year**	MAW	1,711,926	2,416,973	705,047

	HTP	22,380,263	22,181,969	-198,294

	LITP	55,763,283	55,799,317	36,034

**3^rd ^year**	MAW	1,747,500	2,642,079	894,579

	HTP	20,796,602	20,476,162	-320,440

	LITP	56,593,400	56,590,723	-2,677

**Table 5:** Results by Autonomous Region

Region	Eligible population	Scenario without B/N combination Budget impact (€)			Scenario with B/N combination Budget impact (€)			Difference (€) with B/N vs. without B/N		
		
		_1_^st^	_2_^nd^	_3_^rd^	_1_^st^	_2_^nd^	_3_^rd^	_1_^st^	_2_^nd^	_3_^rd^
**Andalucia**	**17,637**	19,440,146	18,100,409	17,937,671	19,626,710	18,223,440	18,067,201	186,563	123,031	129,530

**Aragon**	**1,362**	1,501,246	1,397,786	1,385,219	1,515,653	1,407,287	1,395,222	14,407	9,501	10,003

**Asturias**	**3,289**	3,625,256	3,375,418	3,345,070	3,660,047	3,398,361	3,369,225	34,791	22,943	24,155

**Baleares**	**2,541**	2,800,783	2,607,764	2,584,318	2,827,662	2,625,490	2,602,980	26,879	17,725	18,662

**Canary Islands**	**5,063**	5,580,624	5,196,029	5,149,313	5,634,180	5,231,348	5,186,497	53,556	35,318	37,184

**Cantabria**	**795**	876,278	815,888	808,553	884,688	821,434	814,392	8,409	5,546	5,839

**Castilla La Mancha**	**2,226**	2,453,579	2,284,488	2,263,948	2,447,125	2,300,016	2,280,297	23,547	15,528	16,348

**Castilla León**	**4,197**	4,626,087	4,307,275	4,268,549	4,670,483	4,336,553	4,299,373	44,396	29,277	30,824

**Catalonia**	**7,922**	8,731,918	8,130,149	8,057,052	8,815,717	8,185,411	8,115,233	83,799	55,262	58,181

**Extremadura**	**1,683**	1,855,064	1,727,221	1,711,691	1,872,867	1,738,961	1,724,052	17,803	11,740	12,360

**Galicia**	**7,822**	8,621,694	8,027,522	7,955,347	8,704,435	8,082,086	8,012,794	82,741	54,564	57,447

**Madrid**	**9,606**	10,588,084	9,858,396	9,769,761	10,689,696	9,925,405	9,840,309	101,612	67,009	70,549

**Murcia**	**2,021**	2,227,620	2,074,101	2,055,453	2,248,998	2,088,199	2,070,296	21,378	14,098	14,843

**Navarra**	**799**	880,687	819,994	812,621	889,139	825,567	818,489	8,452	5,574	5,868

**Basque Country**	**2,633**	2,902,189	2,702,182	2,677,887	2,930,041	2,720,549	2,697,224	27,852	18,367	19,337

**La Rioja**	**762**	839,904	782,021	774,990	847,965	787,337	780,587	8,060	5,315	5,596

**Valencia**	**6,425**	7,081,870	6,593,816	6,534,532	7,149,833	6,638,635	6,581,718	67,963	44,819	47,187

**Ceuta**	**603**	664,649	618,844	613,280	671,027	623,050	617,708	6,379	4,206	4,429

**Melilla**	**425**	468,451	436,167	432,245	472,946	439,131	435,367	4,496	2,965	3,121

Total cost of AOT per patient was estimated at €1,102; €1,026 and €1,017 for the first, second and third year, respectively, in the scenario without B/N combination. Incremental total cost per patient comparing the inclusion of the B/N combination to the scenario where methadone was the only medication available, was €10.58, €6.98 and €7.34 in the first, second and third year, respectively. National budgetary impact by target population group for the 3 study years is shown in Table 4.

### Sensitivity analysis

In the sensitivity analysis, distribution of patients in HTP or LITP groups was the most influential parameter on the results. Incremental differences in cost/patient could reach a total of €35.26, €19.13 and €16.46 in the first, second and third year of the study, respectively, assuming that 100% of the population was in HTP group at the beginning of the simulation. On the other hand, if it was to be assumed that 100% of the population was in the LITP group at the beginning of the study, the incremental cost associated to B/N combination compared to a situation where methadone is the sole available AOT would be €0, €1.76 and €3.44 per patient for the first, second and third year, respectively. Detailed results are shown in Table 6.

**Table 6:** Results of one-way sensitivity analyses.

Parameter modified	Value in BC	Value in SA	Scenario without B/N combination	Scenario with B/N combination				Difference (€) with B/N vs. without B/N			
			
			1^st ^year	2^nd ^year	3^rd ^year	1^st ^year	2^nd ^year	3^rd ^year	1^st ^year	2^nd ^year	3^rd ^year
**Transition probabilities**	See table 1	Same values for B/N combination than used for methadone	1,102	1,026	1,017	1,113	1,035	1,028	10.58	9.18	11.13

**B/N combination uptakes**	10%, 15% and 20% for 1^st^, 2^nd ^and 3^rd ^year	0.97%; 2.57% and 3.83% for 1^st^, 2^nd ^and 3^rd ^year	1,102	1,026	1,017	1,103	1,028	1,019	1.03	1.37	1.58

**Initial proportion of patient distribution**	HTP: 30% and LITP: 70%	HTP: 100%	1,477	1,185	1,127	1,513	1,204	1,144	35.260	19.13	16.46
		
		LITP: 100%	941	958	970	941	960	973	0	1.76	3.44

**Resource cost (cost per minute)**	See table 3	+10%	1,201	1,126	1,116	1,218	1,129	1,119	7.78	2.87	2.30
		
		-10%	995	926	918	1,008	937	930	13.38	11.08	12.39

Yearly budgetary impact per patient (€, 2010)

BC: Base Case; SA: Sensitivity analysis

When the number of patients commencing B/N combination from the Murcia Region is applied to national level the incremental total budgetary impact of AOT in the scenario with B/N combination versus scenario without B/N combination was €79.839, €106,513 and €122,872 for the first, second and third year. Differences in cost per patient between the 2 scenarios are €1.03, €1.37 and €1.58 for each of the three years.

## Discussion

The results from the present study show that approval of a novel medication for heroin dependent patients, namely B/N combination compared to the only AOT available, methadone, is associated with increases in direct pharmacological costs, but it is also directly associated with reduction of other type of costs involved, as analysed, including logistics/distribution, production, delivery, supervision and monitoring.

According to the results of the sensitivity analysis, the parameters with the most influence on the final outcomes would be those associated with patient profile and percentage of B/N combination use. The number of patients commencing B/N combination each year, used in the sensitivity analysis, was obtained from a market sales database [36], representing real use of B/N combination in a specific region in Spain (Murcia). Base case is a conservative approach to the approval of B/N combination, as intake values used are higher than real life values. If market conditions of Murcia are extrapolated to national level the total budgetary impact per patient would be €1.03, €1.37 and €1.58 for the first, second and third year of the analysis.

These results are in concordance with those obtained in the previous publication of the original model [24]. The present work is an update as much the current situation in terms of number of patients as costs. Besides, this paper provides more details of the resource consumption and more accurate estimates of the budgetary impact, based on data following the real use of B/N combination.

The model developed for the analysis aims to be a transparent tool. All the parameters included and assumptions considered are presented in such a detailed manner to allow for replication in similar or different settings. Third payer perspective was chosen to provide useful data for the decision makers at the funding administration level.

There are several limitations to be considered in this model, including the very limited number of published reports regarding resource consumption in population in AOT, and as a consequence analysis relied on expert panel estimations. Pragmatic or naturalistic prospective studies conducted under standard clinical practice, designed to collect resources and costs data associated to AOT could provide reliable information to be used in further economic evaluations [37]. A potential limitation of the model refers to the assumption that approval of B/N combination would not cause a significant elevation of the population entering AOT. An increase in the number of patients entering in AOT was reported in the UK when buprenorphine was licensed in the UK in 1999 [38], but early experiences in Spain following with the approval of B/N combination does not suggest that this could be expected [24,30]. Therefore to avoid confounding factors on the final results, it was decided to keep a constant value for population under AOT. Potential improvements in quality of life and benefits resulting from better integration at a social level were not included in the present model, but there is evidence that buprenorphine is associated to better results at psychological, medical, family and work level than other treatments [39]. A conservative approach has been chosen for some aspects. Frequency of urine drug testing remained unchanged with B/N combination, however it would be expected a decrease in number of toxicological screenings, with a subsequent reduction in associated costs [40].

There is the common perception that methadone may be more effective than buprenorphine for as an AOT, primarily based on studies and experiences with low buprenorphine doses and excessively slow induction regimens as used in early buprenorphine trials [11,41]. However subsequent studies showed that the efficacy of buprenorphine sublingual tablet or buprenorphine/naloxone sublingual tablet (Suboxone^®^) is equivalent to that of methadone when sufficient buprenorphine doses, rapid induction, and flexible dosing are used [22,42,43]. Effectiveness has been also tested concluding that buprenorphine is at least as effective as methadone and has a better tolerability profile in reducing illicit opioid when use in clinical practice [8,12,44]. Published evidence have suggested that assuming that B/N combination is not more or less effective than methadone but it will be less expensive in the long run, it is expected that B/N combination would be more cost-effective than methadone when provided to comparable groups of patients [45]. Although methadone will remain an essential relapse prevention pharmacotherapy for opioid addicted individuals, buprenorphine-based regimens may increase access to care and provide safer, more appropriate treatment than methadone for some patients [11,44]. In addition buprenorphine is an alternative treatment for heroin dependent patients, especially for those who do not wish to start or continue with methadone or for those who do not seem to benefit from adequate dosages of methadone [46].

The budget impact analyses are important, along with the cost-effectiveness analysis, as part of a comprehensive economic evaluation of a new health technology [26]. The purpose of a budget impact study is to estimate the financial consequences of adoption and diffusion of a new health-care intervention within a specific health-care setting or system context given inevitable resource constraints [26]. In Spain there are no official threshold values published for health technologies implementation. According to authors' criteria, the increases in total cost of AOT with B/N combination's incorporation obtained in this study seem reasonable, even more if additional benefits at clinical level for patients are considered.

The introduction of new (and expensive) pharmaceutical products is one of the major challenges for health systems [47]. New treatments, procedures and technologies into the services' portfolio of healthcare providers should aim to improve three areas equally: patient access to innovative solutions, the sustainability of the health system and compensation for innovation. Traditional schemes based on fixed prices that fail to consider the product's appropriate use or its results in terms of effectiveness may lead to inefficient decision-making processes [48]. Risk-sharing agreements are defined as a new form of contractual agreement between payers and the pharmaceutical industry for setting the value of an innovation conditional to demonstration of its effectiveness and efficiency in real life [49]. This type of agreements could be taken in mind when health authorities face a regulatory decision on drug pricing and reimbursement in a context of uncertainty [50], as they could diminish the impact on the payer's budget for new and existing medicines brought about by either the uncertainty of the value of the medicine and/or the need to work within finite budgets [51].

## Conclusions

Addition of B/N combination would imply a maximum incremental yearly cost of €10.58 per patient compared to scenario only with methadone and would provide additional benefits

The results of this report would help the Regional and National Authorities to perform a better allocation of available resources associated to addiction treatment services.

## Competing interests

This work was supported by an unrestricted research grant sponsored by Reckitt Benckiser. The authors have not transmitted any conflicts of interest, because the concept, design and development of the model have been carried out independently. IO, ES and MAC are PORIB employees a consultant company specialized in economic evaluation of health technologies.

## Authors' contributions

JMR and MAC conceived of the study and performed a general coordination of the project. FGS and JO have made substantial contributions to conception and model design. IO and ES have involved in analysis. JMR, FGS and JO have played key role in acquisition of data and interpretation of the results. IO, ES and MAC validated the assumptions taken in model design, reviewed the results, participating in interpretations of data, and were involved in drafting the manuscript. All the authors have participated sufficiently in the work to take public responsibility for appropriate portions of the content. All of them have reviewed the final version of the manuscript and have given a final permission of the version to be published.
